# Effect of work organization and demands, violence, health problems and job satisfaction in relation to work stress and social support in prison officers: a cross-sectional study; São Paulo, 2019

**DOI:** 10.1590/S2237-96222025v34e20240714.en

**Published:** 2025-08-11

**Authors:** Soraya Geha Gonçalves, Arthur Eumann Mesas, Daiane Suele Bravo, Marcela Maria Birolim, Camilo Molino Guidoni, Elisabete de Fátima Polo de Almeida Nunes, Rosane Paula Nierotka, Renne Rodrigues

**Affiliations:** 1 Universidade Estadual de Londrina, Programa de Pós-Graduação em Saúde Coletiva, Londrina, PR, Brazil; 2 Universidad de Castilla-La Mancha, Centro de Estudos Sociosanitarios, Cuenca, Espanha; 3 Faculdade Guairacá, Departamento de Enfermagem e Odontologia, Guarapuava, PR, Brazil; 4 Universidade Estadual de Londrina, Departamento de Ciência Farmacêuticas, Londrina, PR, Brazil; 5 Universidade Federal da Fronteira Sul, Curso de Medicina, Chapecó, SC, Brazil

**Keywords:** Occupational Stress, Social Support, Working Conditions, Safety, Cross-Sectional Studies, Estrés Laboral, Apoyo Social, Condiciones de Trabajo, Seguridad, Estudios Transversales

## Abstract

**Objective:**

Analyze the effect of work organization and demands, violence, health problems and job satisfaction in relation to work stress and social support in prison officers.

**Methods:**

This is a cross-sectional study with prison police officers from four penitentiaries in São Paulo. The Demand-Control-Support Questionnaire was used and adjusted multinomial regressions were performed to obtain the odds ratio (OR) and 95% confidence interval (95%CI).

**Results:**

A total of 265 prison officers participated in the study, and poor satisfaction levels with temperature (OR 4.88; 95%CI 2.02; 11.81) and ventilation (OR 3.12; 95%CI 1.31; 7.41) were found to be associated with high-demand work. Furthermore, working more than 15 years as a prison officer (OR 2.59; 95%CI 1.33; 5.08), reporting physical violence (OR 2.84; 95%CI 1.02; 7.93), psychological violence (OR 2.93; 95%CI 1.55; 5.53), poor satisfaction level with lighting (OR 2.80; 95%CI 1.11; 7.08), noise (OR 3.94; 95%CI 2.15; 7.21), ventilation (OR 2.15; 95%CI 1.20; 3.85), and furniture (OR 2.92; 95%CI 1.52; 5.60) in the workplace were associated with lower social support.

**Conclusion:**

The worst level of satisfaction with temperature and ventilation was associated with highly demanding work. Furthermore, suffering violence, greater physical demands, and a lower level of satisfaction with workplace conditions were associated with less social support. The need for improvements in infrastructure and in the organization of the work process is highlighted.

## Resumen

**Objetivo:**

Analizar el efecto de la organización y exigencias del trabajo, la violencia, los problemas de salud y la satisfacción laboral en relación con el estrés laboral y el apoyo social en agentes de policía penitenciaria.

**Métodos:**

Se trata de un estudio transversal con agentes de policía penitenciaria de cuatro penitenciarías de São Paulo. Se utilizó el Demand-Control-Support Questionnaire y se realizaron regresiones multinomiales ajustadas para obtener la razón de probabilidades (odds ratio, OR) y el intervalo de confianza del 95% (IC95%).

**Resultados:**

Participaron en el estudio 265 funcionarios de prisiones y se identificó que los bajos niveles de satisfacción con la temperatura (OR 4,88; IC95% 2,02; 11,81) y la ventilación (OR 3,12; IC95% 1,31; 7,41) se asociaron con trabajo de alto esfuerzo. Además, trabajar más de 15 años como agente de policía penitenciario (OR 2,59; IC95% 1,33; 5,08), reportar violencia física (OR 2,84; IC95% 1,02; 7,93), violencia psicológica (OR 2,93; IC95% 1,55; 5,53), bajo nivel de satisfacción con la iluminación (OR 2,80; IC95% 1,11; 7,08), ruido (OR 3,94; IC95% 2,15; 7,21), ventilación (OR 2,15; IC95% 1,20; 3,85) y mobiliario (OR 2,92; IC95% 1,52; 5,60) en el entorno de trabajo se asociaron con un menor apoyo social.

**Conclusión:**

El peor nivel de satisfacción con la temperatura y la ventilación se asoció con trabajos altamente exigentes. Además, sufrir violencia, mayores exigencias físicas y un menor nivel de satisfacción con las condiciones del entorno laboral se asociaron con un menor apoyo social. Se destaca la necesidad de mejoras en la infraestructura y en la organización del proceso de trabajo.

**Ethical aspects**

This research respected ethical principles, having obtained the following approval data:

Research Ethics Committee: Universidade Estadual de Londrina

Opinion number: 2.826.580

Approval date: 16/8/2018

Certificate of Submission for Ethical Appraisal: 87250718.7.3003.5563

Informed Consent Form: Obtained from all participants prior to collection.

## Introduction

The penitentiary system is responsible for, among other duties, managing the enforcement of prison sentences handed down by the courts (1,2), holding a total of 832,295 people in Brazil at the end of 2022, 94.5% of whom are male (2). To this end, in December 2022, this system had more than 120,000 professional prison officers (2) carrying out their activities in a place designed and built so that control and hierarchy are respected, but which is paradoxically unstable, as it depends on the dynamics and relationships between prison officers and persons deprived of liberty (1,3). Thus, the workplace of prison officers is marked by unhealthiness and dangerousness (4,5), a context that can change the way in which working conditions influence occupational stress (6). 

Although there is no consensus (7), when it comes specifically to prison officers, Cullen and collaborators define occupational stress as negative repercussions on mental health, such as psychological suffering (8), resulting from a process in which work demands exceed the workers’ ability to adapt (9). The dynamics are affected by other variables, with emphasis on the control that workers can (or cannot) exercise over their work process (9) and social support, which can mitigate the effects of work demands (10). 

Due to its importance, occupational stress, coexistence of greater work demands and less control over this process, commonly called high-strain work (11,12), has been associated with psychiatric symptoms, emotional exhaustion, mental suffering, and worse professional achievement (13). The possible mechanisms for this association arise from psychological changes (fear, tension, and anxiety), physiological changes (changes in cortisol, catecholamines, and interleukin-6), psychosomatic changes (sleep disorders, headache, and fatigue), and changes in the immune, cardiovascular and metabolic systems, which can increase the chances of disease and mortality (14,15). 

Considering the complexity of the topic and the impacts on workers’ health, in addition to harm reduction actions (5), measures are needed to identify which work factors influence high-demand work. Thus, high-demand work is a dependent variable in studies (5,6,16). However, most studies conducted on occupational stress with prison officers happen in countries with prison system conditions that are very different from those in Brazil, such as the United States and European nations (5). In these countries, high exposure to violence, work overload and role conflict between custody and rehabilitation are emphasized as plausible causes of occupational stress, and burnout, anxiety, and cardiovascular diseases as possible repercussions on the health of prison officers (5).

In Brazil, studies focus mainly on the psychosocial impacts of stress, precarious working conditions, and professional stigma. The findings of this study reinforce work overload, job insecurity and lack of institutional support as risk factors. Unlike what happens in other countries, systemic negligence and the absence of public policies aimed at the mental health of prison officers stand out in Brazil (5). Added to this context is the fact that, in Brazil, 83.2% of workers in the prison system are male (2), that is, these are individuals who, for the most part, have less access to health services (17), and, therefore, could benefit more from the identification of factors that may increase work-related stress and strategies that aim to reduce its effects on workers’ health. 

Given this panorama, and considering the importance of this professional category and the need for a better understanding of occupational variables in highly demanding work, this study aims to analyze the effect of work organization and demands, violence, health problems and job satisfaction in relation to work stress and social support in prison police officers. 

## Method

### Study design

This is a cross-sectional study, conducted between January and August 2019. 

### Context 

The study was conducted in four penitentiaries in the western region of the state of São Paulo, in the municipalities of Assis, Florínea, Martinópolis and Paraguaçu Paulista. The research was part of the “AGEPEN Study: Working conditions, mental health and sleep among prison officers in the State of São Paulo” (18,19), which selected, for convenience, four penitentiaries of similar size, intended exclusively for male individuals deprived of liberty, to conduct the study with these professionals.

### Participants 

The survey inclueded all prison officers who met the inclusion criteria: being male and having worked for six months or more as prison officers in the units surveyed. Prison officers who were not interviewed and those who presented incomplete records on the study variables were considered as losses. For the study, individuals with less than 12 months of experience in the profession were excluded from data analysis. Data collection took place throughout the working day, with an explanation of the research objectives being given to all teams and shifts at the start of the workday, and then, for those who agreed to participate, the Informed Consent Formulary was given and explained. The prison officers who signed and returned the form received a structured anonymous questionnaire, along with a brown envelope, in which they had to deposit the completed material, which was collected at the end of the shift.

### Variables 

The sociodemographic characterization was composed of age (continuous), education (dichotomized into high school; and higher education - completed higher education, postgraduate or master’s/doctorate) and cohabitation (lives with a partner: married or in a stable union; lives without a partner: single, widowed or divorced). 

The independent variables were the occupational aspects: years of work as a prison officer (continuous and categorized as ≤15 years and >15 years; dichotomized based on the median), the work sector (dichotomized into administrative: without contact with persons deprived of liberty [administrative, IT, human resources, among other administrative areas]; or operational: with contact [gatekeeping, prison cell, reception, discipline, fleet, among other sectors in contact with persons deprived of liberty]), work shift in the penitentiary (daily worker 8h/day; night shift worker 12h/36h; or daily shift worker 12h/36h), mental and physical demands (low/moderate or high), health conditions (yes/no) related to work as a prison officer (any illness, accident, injury or other health problem) in the 12 months preceding the survey, having suffered in the 12 months preceding the survey any psychological violence (teasing or humiliating/embarrassing situations, moral harassment or threat to physical or family integrity) or physical violence (physical aggression, with sharp or firearm weapons) (yes/no), and the degree of satisfaction with working conditions – temperature, lighting, noise, ventilation, hygiene and furniture (dichotomized as bad; or regular/good). 

### Data sources and measurement

Occupational stress was measured using the Demand-Control-Support Questionnaire (20). The validated version with adequate psychometric properties in Portuguese, used in the research, has 17 questions, five to assess psychological demand at work, six to assess control over work and six to assess social support (20). In the present study, the quadrant analysis was performed by adding the scores of each dimension, and subsequently dividing them into two categories based on the median (11,20,21). Thus, cut-off points were values greater than 14 as high demand, greater than 15 points as self-control at work, and greater than 17 points as high social support. The results obtained were combined as recommended as the four categories of the demand-control model: high demand (high demand and low control), active work (high demand and high control), passive work (low demand and low control) and low demand (low demand and high control). For the analysis, the reference group adopted was the low-demand work group. The dimension of *social support* was dichotomized into low and high, with high social support being considered as the reference category.

### Bias control

The formulation of the research questionnaire was preceded by checking the literature for validated instruments and questions for inclusion in the research, such as the Demand-Control-Support Questionnaire. Questions that were not extracted from validated instruments were assessed by a committee of epidemiology experts, composed of two doctoral students in public health and five professors, all with doctorates in Public Health. The definitive version was approved by consensus by the experts committee.

Regarding the collection strategies, this was preceded by a pilot study in a prison unit, in which these strategies were improved, so that the prison officers could be assured as to the confidentiality and anonymity of the research. The instruments were entered in duplicate using the Epi Info 3.5.4 software, with subsequent consolidation and correction of any inconsistencies. Finally, statistical analyses were adjusted for possible confounding variables, as detailed in the statistical methods.

### Study size and sample

The study did not provide an estimate for sample size, as it sought to interview all prison officers in the included penitentiaries. During the study period, a total of 566 prison officers were registered in the four penitentiaries included. Of these, 213 (37.6%) were considered losses (82 were on leave, 36 prison officers could not be located after three attempts, and 95 refused to participate in the study), resulting in a response rate of 62.4% (n=353). Furthermore, 87 (15.4%) prison officers left one or more variables in the study incomplete, and were therefore considered losses, and one had been in the profession for less than 12 months, and was excluded from the analyses, resulting in a study sample of 265 (46.8%) prison officers. 

### Statistical methods

The analyses were performed using the Statistical Package for the Social Science software, version 25.0. Absolute and relative frequencies (categorical variables) were presented, as well as mean and standard deviation (for age). Aiming to identify possible associations that could alter the analyses of occupational factors and occupational stress, an assessment was conducted of the possible association of the characterization variables in relation to the dependent variables. The chi-square test was used for categorical variables, the Kruskal-Wallis test for the demand-control quadrant and the Mann-Whitney test for the dichotomization of social support (continuous variables with non-parametric distribution according to the Shapiro-Wilk test). Additionally, Spearman’s correlation test was performed between the characterization and occupational variables in relation to the sum of the dimensions of demand, control, and social support. For this analysis, age in years (continuous variable) was considered, while categorical variables were evaluated based on the numbers 1 and 2, being for education secondary education and higher education, and for cohabitation, without a partner and with a partner, respectively.

The effect of occupational variables on occupational stress was assessed using multinomial logistic regression models in relation to the demand-control and social support model, with calculation of the odds ratios (OR) value and 95% confidence intervals (95%CI). Crude models (not shown) and adjusted models were performed for variables that have an effect in the literature (age, education) (5) or in the present research (cohabitation) with the dependent variables, as well as the inclusion of social support (continuous) as an adjustment for the analyses in which the demand-control model was the dependent variable, and the demand-control model as an adjustment for the analyses in which social support was the dependent variable (16,20). 

## Results

The general profile of the participating prison officers identified that the majority were 45 years old or younger (54.7%), had a partner (86.8%) and had a secondary level of education (55.5%). The mean age was 44.5 ± 7.9 years, with no difference in age distribution among participants when stratified according to the demand-control model (p-value 0.223) or social support (p-value 0.487). Regarding distribution of the quadrants, 24.5% of prison officers were classified as high-demand work; 30.9%, passive work; 20.8%, active work; and 23.8%, low-demand work. Regarding social support, 55.8% of participants scored ≤17 and were classified as having lower social support (Table 1). 

**Table 1:** Absolute (n) and relative (%) frequencies, and results of the chi-square test of sociodemographic characteristics by study variables. São Paulo, 2019 (n=265)

		Demand-control model					Social support		
Variables	Total n	High demand n (%)	Passive n (%)	Active n (%)	Low demand n (%)	p-value	Low n (%)	High n (%)	p-value
Education						0.106			0.441
High school	147	33 (22.4)	47 (32,0)	25 (17.0)	42 (28.6)		79 (53.7)	68 (46.3)	
Higher education	118	32 (27.1)	35 (29.7)	30 (25.4)	21 (17.8)		69 (58.5)	49 (41.5)	
Cohabitation						0.228			0.002
Without a partner	35	4 (11.4)	13 (37.2)	7 (20.0)	11 (31.4)		11 (31.4)	24 (68.6)	
With a partner	230	61 (26.5)	69 (30.0)	48 (20.9)	52 (22.6)		137 (59.6)	93 (40.4)	

Among the sociodemographic variables investigated, having a partner showed a higher frequency of individuals classified as having lower social support (Table 1) and an effect compatible with the decrease in the continuous score of social support (Table 2). Increasing age had an effect compatible with a decrease in the work demand score, while having higher education had an effect compatible with an increase in the work demand score and a decrease in the social support score (Table 2).

**Table 2:** Spearman correlation to estimate the correlation coefficient (rho) and p-value, between sociodemographic variables and continuous score of the dimensions demand, control, and social support. São Paulo, 2019 (n=265)

Variables	Age Rho; p-value	Education Rho; p-value	Cohabitation Rho; p-value	Demand Rho; p-value	Control Rho; p-value
Age	-				
Education	-0.112; 0.069	-			
**Cohabitation**	0.006; 0.917	0.036; 0.565	-		
Demand	-0.136; 0.027	0.145; 0.019	0.117; 0.057	-	
Control	-0.022; 0.719	-0.050; 0.418	-0.063; 0.310	0.115; 0.060	-
Support	-0.018; 0.770	-0.126; 0.040	-0.230; <0.001	-0.271; <0.001	0.289; <0.001

Prison officers who rated their level of satisfaction with the temperature and ventilation of their workplace as poor showed a higher frequency of highly demanding work (Table 3), with a correlation between the poor level of satisfaction and the lowest score in the control over work score (Table 2). The lowest level of social support was observed mainly in individuals with more than 15 years in this occupation, who reported having suffered physical and psychological violence and who evaluated the level of satisfaction with the conditions of the workplace as poor.

**Table 3:** Absolute (n) and relative (%) frequencies of occupational characteristics by study variables. São Paulo, 2019 (n=265)

	Demand-control model					Social support	
Variables	High demand n (%)	Passive n (%)	Active n (%)	Low demand n (%)		Low n (%)	High n (%)
**Years of experience as a prison officer**							
≤15 years	29 (25.4)	35 (30.7)	24 (21.1)	26 (22.8)		54 (47.4)	60 (52.6)
>15 years	36 (23.9)	47 (31.1)	31 (20.5)	37 (24.5)		94 (62.3)	57 (37.7)
**Work sector**							
Operational	51 (26.2)	65 (33.3)	36 (18.4)	43 (22.1)		113 (57.9)	82 (42.1)
Administrative	14 (20.0)	17 (24.3)	19 (27.1)	20 (28.6)		35 (50.0)	35 (50.0)
**Work shift**							
Night shift duty	23 (26.7)	33 (38.4)	4 (4,7)	26 (30.2)		49 (57.0)	37 (43.0)
Day shift duty	28 (23.5)	34 (28.6)	26 (21.8)	31 (26.1)		66 (55.5)	53 (44.5)
Daily worker	14 (23.3)	15 (25.0)	25 (41.7)	6 (10.0)		33 (55.0)	27 (45.0)
**Physical demands**							
High	57 (28.7)	49 (24.6)	52 (26.1)	41 (20.6)		119 (59.8)	80 (40.2)
Low/moderate	8 (12,1)	33 (50.0)	3 (4.5)	22 (33.4)		29 (43.9)	37 (56.1)
**Mental demands**							
High	13 (36.1)	2 (5,5)	11 (30.6)	10 (27.8)		22 (61.1)	14 (38.9)
Low/moderate	52 (22.8)	80 (34.9)	44 (19.2)	53 (23.1)		126 (55.0)	103 (45.0)
**Work-related health condition**							
Yes	17 (34.7)	13 (26.5)	9 (20.4)	10 (20.4)		31 (63.3)	18 (36.7)
No	48 (22.2)	69 (31.9)	46 (21.3)	53 (24.6)		117 (54.2)	99 (45.8)
**Psychological Violence**							
Yes	26 (32.5)	17 (21.3)	23 (28.7)	14 (17.5)		60 (75.0)	20 (25.0)
No	39 (21.1)	65 (35.1)	32 (17.3)	49 (26.5)		88 (47.6)	97 (52.4)
**Physical Violence**							
Yes	9 (36.0)	5 (20.0)	4 (16,0)	7 (28.0)		19 (76.0)	6 (24.0)
No	56 (23.3)	77 (32.1)	51 (21.3)	56 (23.3)		129 (53.8)	111 (46.2)
**Satisfaction with the workplace temperature**							
Bad	35 (35.4)	37 (37.4)	16 (16.2)	11 (11.0)		67 (67.7)	32 (32.3)
Good/average	30 (18.1)	45 (27.1)	39 (23.5)	52 (31.3)		81 (48.8)	85 (51.2)
**Satisfaction with workplace lighting**							
Bad	15 (41.7)	11 (30.6)	7 (19.4)	3 (8,3)		29 (80.6)	7 (18.4)
Good/average	50 (21.8)	71 (31.0)	48 (21.0)	60 (26.2)		119 (52.0)	110 (48.0)
**Satisfaction with the noise level in the workplace**							
Bad	32 (32.7)	35 (35.7)	15 (15,3)	16 (16.3)		76 (77.6)	22 (22.4)
Good/average	33 (19.8)	47 (28.1)	40 (24.0)	47 (28.1)		72 (43.1)	95 (56.9)
**Satisfaction with ventilation in the workplace**							
Bad	32 (32.3)	36 (36.4)	20 (20.2)	11 (11.1)		71 (71.7)	28 (28.3)
Good/average	33 (19.9)	46 (27.7)	35 (21.1)	52 (31.3)		77 (46.4)	89 (53.6)
**Satisfaction with the hygiene of the workplace**							
Bad	17 (34.7)	14 (28.6)	10 (20.4)	8 (16.3)		36 (73.5)	13 (26.5)
Good/average	48 (22.2)	68 (31.5)	45 (20.8)	55 (25.5)		112 (51.9)	104 (48.1)
**Satisfaction with workplace furniture**							
Bad	27 (34.6)	26 (33.3)	16 (20.6)	9 (11.5)		61 (78.2)	17 (21.8)
Good/average	38 (20.3)	56 (29.9)	39 (20.9)	54 (28.9)		87 (46.5)	100 (53.5)

The adjusted models identified that only a poor assessment of satisfaction with temperature and ventilation had an effect compatible with an increase in high-demand work (Table 4). Lower social support was associated with longer time as a prison officer (more than 15 years), reports of psychological and physical violence, and a poor level of satisfaction with lighting, noise, ventilation, and furniture in the workplace (Table 5).

**Table 4:** Adjusted odds ratios (OR) and 95% confidence intervals (95%CI) of the demand–control model for the study variables. São Paulo, 2,019 (n=265)

Variables	High demand OR (95%CI)	Passive OR (95%CI)	Active OR (95%CI)
**Years of experience as a prison officer**			
≤15 years	1.00	1.00	1.00
>15 years	0.57 (0.22; 1.49)	0.62 (0.26; 1.49)	1.06 (0.40; 2.81)
**Work sector**			
Operational	1.54 (0.66; 3.59)	1.69 (0.77; 3.68)	0.89 (0.40; 1.98)
Administrative	1.00	1.00	1.00
**Work shift**			
Night shift duty	0.36 (0.11; 1.20)	0.48 (0.16; 1.47)	0.04 (0.01; 0.15)
Day shift duty	0.31 (0.09; 1.00)	0.39 (0.13; 1.21)	0.18 (0.06; 0.53)
Daily worker	1.00	1.00	1.00
**Physical demands**			
High	2.41 (0.91; 6.38)	0.56 (0.26; 1.18)	7.41 (2.01; 27.38)
Low/moderate	1.00	1.00	1.00
**Mental demands**			
High	1.27 (0.46; 3.46)	0.12 (0.02; 0.59)	1.40 (0.53; 3.74)
Low/moderate	1.00	1.00	1.00
**Work; related health condition**			
Yes	1.37 (0.53; 3.52)	0.80 (0.31; 2.05)	0.84 (0.30; 2.33)
No	1.00	1.00	1.00
**Psychological Violence**			
Yes	1.03 (0.43; 2.47)	0.52 (0.22; 1.25)	1.78 (0.76; 4.21)
No	1.00	1.00	1.00
**Physical Violence**			
Yes	0.99 (0.31; 3.14)	0.45 (0.13; 1.56)	0.48 (0.13; 1.83)
No	1.00	1.00	1.00
**Satisfaction with the temperature of the workplace**			
Bad	4.88 (2.02; 11.81)	3.80 (1.67; 8.66)	1.86 (0.74; 4.63)
Good/average	1.00	1.00	1.00
**Satisfaction with the lighting in the workplace**			
Bad	3.83 (0.97; 15.11)	2.53 (0.65; 9.89)	2.21 (0.52; 9.34)
Good/average	1.00	1.00	1.00
**Satisfaction with the noise level in the workplace**			
Bad	1.55 (0.67; 3.53)	1.55 (0.72; 3.33)	0.83 (0.34; 2.01)
Good/average	1.00	1.00	1.00
**Satisfaction with ventilation in the workplace**			
Bad	3.12 (1.31; 7.41)	3.07 (1.36; 6.89)	2.23 (0.92; 5.39)
Good/average	1.00	1.00	1.00
**Satisfaction with the hygiene of the workplace**			
Bad	1.63 (0.59; 4.45)	1.13 (0.42; 3.00)	1.40 (0.49; 4.04)
Good/average	1.00	1.00	1.00
**Satisfaction with workplace furniture**			
Bad	2.30 (0.91; 5.86)	1.54 (0.84; 2.85)	1.88 (0.72; 4.92)
Good/average	1.00	1.00	1.00

**Table 5:** Adjusted odds ratios (OR) and 95% confidence intervals (95%CI) of the *social support* dimension by the study variables. São Paulo, 2,019 (n=265)

Variables	Low social support OR (95%CI)
**Years of experience as a prison officer**	
≤15	1.00
>15	2.59 (1.33; 5.08)
**Work sector**	
Operational	1.18 (0.65; 2.17)
Administrative	1.00
**Work shift**	
Night shift duty	1.42 (0.66; 3.08)
Day shift duty	1.21 (0.61; 2.43)
Daily worker	1.00
**Physical demands**	
High	1.64 (0.85; 3.15)
Low/moderate	1.00
**Mental demands**	
High	1.11 (0.50; 2.47)
Low/moderate	1.00
**Work-related health condition**	
Yes	1.09 (0.54; 2.20)
No	1.00
**Psychological Violence**	
Yes	2.93 (1.55; 5.53)
No	1.00
**Physical Violence**	
Yes	2.84 (1.02; 7.93)
No	1.00
**Satisfaction with the temperature of the workplace**	
Bad	1.69 (0.94; 3.03)
Good/average	1.00
**Satisfaction with the lighting in the workplace**	
Bad	2.80 (1.11; 7.08)
Good/average	1.00
**Satisfaction with noise level in the workplace**	
Bad	3.94 (2.15; 7.21)
Good/average	1.00
**Satisfaction with ventilation in the workplace**	
Bad	2.15 (1.20; 3.85)
Good/average	1.00
**Satisfaction with the hygiene of the workplace**	
Bad	1.89 (0.90; 3.94)
Good/average	1.00
**Satisfaction with workplace furniture**	
Bad	2.92 (1.52; 5.60)
Good/average	1.00

## Discussion

Among the occupational variables investigated, evaluating the level of satisfaction with the temperature and ventilation of the workplace as poor had an effect compatible with the increased chance of highly demanding and passive work. Having greater mental demands had an effect compatible with a lower chance of passive work. Compared to prison officers who worked daily (8 hours a day), those on night and day shifts (12 hours) showed an effect compatible with a lower chance of active work. Reporting greater physical demands at work had an effect compatible with a greater chance of active work. Finally, prison officers with more than 15 years of experience, reports of physical and psychological violence, and those with a poor level of satisfaction regarding some conditions in the workplace showed an effect compatible with a greater chance of having less social support

In relation to work, the cut-off point for high demand (˃14) was close to that found for police officers (˃14) (22), administrative technicians (˃14) (23) and teachers (˃15) (16, 23). The cutoff point for low job control (≤15) was slightly higher than that observed in other law enforcement professionals (≤11 police officers) (22) and lower than that reported in professionals in the educational field. (≤17) (16). That way, We can infer that, although the organization of work is different between these professions, prison officers evaluate the workload in a comparable way to that perceived by other professionals. However, control over the work process itself appears to be more limited among prison officers, possibly due to its nature, organized into ascending levels of command and with risks inherent in professional practice (1). 

Working on a shift basis, regardless of the shift, had an effect compatible with a lower chance of active work. These findings can be explained, even if in part, by the theory of the “imprisonment” effect (5), according to which continuous exposure, without the possibility of rest the day after work, as occurs with those who work on-call, can intensify the effects of the workload on mental health. According to this theory, working on a shift basis with a day off the next day, allows a greater disconnect from work activities and the recovery from the effects generated by work demands.

The association found between highly demanding work and a lower level of job satisfaction may be due to the fact that this work condition, in which there is an exacerbation of the negative effects of demands, generates a negative evaluation by the professional regarding their work process and future perspectives in relation to their working life, which may lead to a feeling of inability to perform their functions (24). In this context, in several prison institutions the influence of occupational stress on the reduction of job satisfaction is identified (25). Although these effects are well described in the literature (26), in the present study only the poor satisfaction level with temperature and lighting showed an effect compatible with greater occupational stress. A possible explanation for this finding is the approach adopted in the study, which individually analyzed each aspect of the workplace, making it possible to identify the factors that had the greatest impact on the perception of the prison officers investigated.

The organization of the work process in penitentiaries can, at least in part, justify the association between high physical demands and active work since physical work is mostly conducted by prison officers in operational sectors. This characteristic means that these prison officers, while carrying out more physical activities, are also more exposed to persons deprived of liberty, which can accentuate the perception of the demands of the job (5,24). This exposure may also provide greater control over how they perform their duties, in contrast to correctional officers in administrative roles. Although the work sector has a strong influence on working hours, another important aspect also linked to this variable is the level of exposure to conflict situations, the need to perform a dual function (education and maintenance of order) and greater contact with persons deprived of liberty, which can increase occupational stress (27,28).

The perception of greater mental demands had an effect compatible with a lower chance of classification as passive work. This association seems to be justified, once again, by the “imprisonment” effect (5), aggravated by the characteristics of the work process, in which prison officers in coordination roles are inserted into a well-established routine, which does not provide the possibility of adapting the way they work (29). However, no studies were found that have focused on the organization of work and functions that prison officers perform and the effects of these variables on the demand and control of the work process.

Regarding social support, the effects identified seem to point in the direction that prison officers with less social support feel the physical demands, violence, and conditions of the workplace more intensely. This confirms the mitigating role that social support has, both in the face of work stress (high demands and low control) and other more general occupational variables (16,20,30). These findings justify the apparent greater exhaustion identified in prison officers, compared to other professions, making it necessary, in addition to the analysis of occupational stress, to consider the interference of other individual variables, such as sex and age, as well as variables related to work organization, such as working hours, trauma and performance of functions with greater exposure to conflicts (28,31).

A number of theories try to explain the mechanisms involved in this process, and, in general terms, they postulate that greater social support can directly reduce the effect of instrumental work demands, emotional conflicts and conflicts related to work and personal life balance (30). Furthermore, social support has indirect effects, increasing resilience and other coping mechanisms for stressful situations (32). These effects end up spreading across the network, generating a reduction in occupational stress and burnout, in addition to assisting with individual resources, such as autonomy and work skills, which together can improve autonomy and engagement at work, promoting better results, which feed back into the chain through improved social support (30). 

However, reducing occupational stress transcends the dimensions of mental health, influencing other behaviors that are important for a healthy lifestyle, such as sleep (18) and diet (33), which reinforces the importance of actions aimed at controlling or mitigating it. Thus, increasing social support has proven to be an efficient measure for reducing occupational stress and improving quality of life (34). Due to the complexity of the problem, there is a need to adopt systematic changes, given that specific changes to improve relationships and hierarchy have a minor impact on occupational stress, and such changes must be accompanied by a reduction in workload (35).

This study has as its main limitations the cross-sectional design, which does not allow the assessment of causality or directionality between the variables investigated, as well as the fact that the data relate only to prison officers of one sex (male) and from only one Federation Unit, which may limit the generalization of the findings to other populations. Furthermore, the extrapolation of results is considered limited, even for the penitentiaries included in the research, due to the high percentage of losses. However, the study’s strengths include the use of internationally validated scales and, despite the losses, the high number of prison officers who responded to all the research questions, which provided a comprehensive study with statistical analyses adjusted for confounding factors. 

Based on the findings, the importance of strategies that can promote greater control over work, reduce demands, and strengthen social support networks among professionals is reinforced as a way of mitigating the adverse health impacts caused by occupational stress.

## Data Availability

The database used in the research can be obtained by sending a request and justification to the corresponding author.
