# Supplementary material for: Effect of work organization and demands, violence, health problems and job satisfaction in relation to work stress and social support in prison officers: a cross-sectional study; São Paulo, 2019
Source: Epidemiol Serv Saude. 2025 Aug 11;34:e20240714. doi: 10.1590/S2237-96222025v34e20240714.en (PMC12342845; doi:10.1590/S2237-96222025v34e20240714.en)
Supplement: Translation

# Translation: Efeito da organização e exigências do trabalho, violências, problemas de saúde e satisfação no trabalho em relação ao estresse laboral e apoio social em policiais penais: estudo transversal, São Paulo, 2019

**DOI:** 10.1590/S2237-96222025v34e20240714.pt

**Authors:** Soraya Geha Gonçalves, Arthur Eumann Mesas, Daiane Suele Bravo, Marcela Maria Birolim, Camilo Molino Guidoni, Elisabete de Fátima Polo de Almeida Nunes, Rosane Paula Nierotka, Renne Rodrigues

**Affiliations:** 1 Universidade Estadual de Londrina, Programa de Pós-Graduação em Saúde Coletiva, Londrina, PR, Brasil; 2 Universidad de Castilla-La Mancha, Centro de Estudos Sociosanitarios, Cuenca, Espanha; 3 Faculdade Guairacá, Departamento de Enfermagem e Odontologia, Guarapuava, PR, Brasil; 4 Universidade Estadual de Londrina, Departamento de Ciência Farmacêuticas, Londrina, PR, Brasil; 5 Universidade Federal da Fronteira Sul, Curso de Medicina, Chapecó, SC, Brasil

**Keywords:** Estresse Ocupacional, Apoio Social, Condições de Trabalho, Segurança, Estudos Transversais

## Abstract

**Objetivo:**

Analisar o efeito da organização e exigências do trabalho, violências, problemas de saúde e satisfação no trabalho em relação ao estresse laboral e apoio social em policiais penais.

**Métodos:**

Trata-se de um estudo transversal, com policiais penais de quatro penitenciárias de São Paulo. Foi empregado o Demand-Control-Support Questionnaire e realizadas regressões multinomial ajustadas para obtenção da razão de chances (*odds ratio*, OR) e intervalo de confiança de 95% (IC95%).

**Resultados:**

Participaram do estudo 265 policiais penais, sendo identificado que a nível de satisfação ruim com temperatura (OR 4,88; IC95% 2,02; 11,81) e com ventilação (OR 3,12; IC95% 1,31; 7,41) apresentaram associação com trabalho de alta exigência. Além disso, trabalhar mais de 15 anos como policial penal (OR 2,59; IC95% 1,33; 5,08), relato de violência física (OR 2,84; IC95% 1,02; 7,93), violência psicológica (OR 2,93; IC95% 1,55; 5,53), nível de satisfação ruim com iluminação (OR 2,80; IC95% 1,11; 7,08), ruído (OR 3,94; IC95% 2,15; 7,21), ventilação (OR 2,15; IC95% 1,20; 3,85) e mobília (OR 2,92; IC95% 1,52;5,60) do ambiente de trabalho se associaram com menor apoio social.

**Conclusão:**

O pior nível de satisfação com a temperatura e a ventilação esteve associada a um trabalho de alta exigência. Ainda, sofrer violência, maiores demandas físicas e o pior nível de satisfação com as condições do ambiente de trabalho se associaram a um menor apoio social. Ressalta-se a necessidade de melhorias na infraestrutura e na organização do processo de trabalho.

**Aspectos éticos**

A presente pesquisa respeitou os princípios éticos, obtendo os seguintes dados de aprovação:

Comitê de ética em pesquisa: Universidade Estadual de Londrina

Número do parecer: 2.826.580

Data de aprovação: 16/8/2018

Certificado de apresentação de apreciação ética: 87250718.7.3003.5563

Registro do consentimento livre e esclarecido: Obtido de todos os participantes antes da coleta.

## Introdução

O sistema penitenciário é responsável por, entre outras atribuições, gerir o cumprimento das penas de reclusão sentenciadas pela Justiça (1,2), custodiando no Brasil, ao final de 2022, um total de 832.295 pessoas, 94,5% destes do sexo masculino (2). Para tanto, este sistema contava, em dezembro de 2022, com mais de 120 mil policiais penais (2) profissionais realizando suas atividades em um local projetado e construído para que o controle e a hierarquia sejam respeitados, mas que é paradoxalmente instável, pois depende da dinâmica e das relações dos policiais penais e das pessoas privadas de liberdade (1,3). Desse modo, o local de trabalho dos policiais penais é marcado pela insalubridade e periculosidade (4,5), contexto esse que pode alterar o modo como as condições de trabalho influenciam o estresse ocupacional (6). 

Embora não exista consenso (7), em se tratando especificamente de policiais penais, Cullen e colaboradores definem o estresse ocupacional como repercussões negativas sobre a saúde mental, a exemplo o sofrimento psicológico (8), resultantes de um processo em que as demandas do trabalho extrapolam a capacidade de adaptação dos trabalhadores (9). A dinâmica é afetada por outras variáveis, com destaque para o controle que os trabalhadores podem (ou não) exercer sobre o seu processo de trabalho (9) e para o apoio social, que pode atenuar os efeitos das demandas de trabalho (10). 

Devido a sua importância, o estresse ocupacional, coexistência de maiores demandas de trabalho e menor controle sobre este processo, comumente denominado de trabalho de alta exigência (11,12), tem sido associado a sintomas psiquiátricos, exaustão emocional, sofrimento mental e pior realização profissional (13). O possível mecanismos para essa associação decorre de alterações psicológicas (medo, tensão e ansiedade), fisiológicas (alterações no cortisol, catecolaminas e interleucinas-6), psicossomáticas (distúrbios do sono, cefaleia e fadiga), e dos sistemas imunológico, cardiovascular e metabólico, que podem aumentar as chances do surgimento de doenças e mortalidade (14,15). 

Considerando-se a complexidade do tema e os impactos para a saúde dos trabalhadores, além de ações de redução de danos (5), fazem-se necessárias ações de identificação de quais fatores laborais influenciam o trabalho de alta exigência, tendo, desta forma, o trabalho de alta exigência como variável dependente nos estudos (5,6,16). No entanto, a maioria dos estudos realizados a respeito do estresse ocupacional com policiais penais são realizados em países com condições do sistema carcerário muito distintas das brasileiras, como nos Estados Unidos e em nações da Europa (5). Nesses países, enfatiza-se a alta exposição à violência, a sobrecarga de trabalho e o conflito de papéis entre custódia e reabilitação como possíveis causas do estresse ocupacional, e burnout, ansiedade e doenças cardiovasculares como possíveis repercussões sobre a saúde dos policiais penais (5).

No Brasil, os estudos focam principalmente os impactos psicossociais do estresse, condições precárias de trabalho e estigma profissional. Os achados reforçam a sobrecarga, insegurança no emprego e falta de suporte institucional como fatores de risco. Diferentemente do cenário internacional, destaca-se a negligência sistêmica e a ausência de políticas públicas voltadas à saúde mental dos policiais penais (5). Soma-se a esse contexto o fato de que, no Brasil, 83,2% dos trabalhadores do sistema penitenciário são do sexo masculino (2), ou seja, são indivíduos que em sua maioria possuem menor acesso aos serviços de saúde (17), e, portanto, poderiam se beneficiar mais da identificação dos fatores que possam aumentar o estresse laboral e de estratégias que visem diminuir seus efeitos sobre a saúde dos trabalhadores. 

Diante deste panorama, considerando a importância desta categoria profissional e da necessidade de melhor entendimento das variáveis ocupacionais no trabalho de alta exigência, este estudo tem como objetivo analisar o efeito da organização e exigências do trabalho, violências, problemas de saúde e satisfação no trabalho em relação ao estresse laboral e apoio social em policiais penais. 

## Método

### Delineamento

Trata-se de um estudo transversal, realizado entre janeiro e agosto de 2019. 

### Contexto 

O estudo foi realizado em quatro penitenciárias da região oeste do estado de São Paulo, nos municípios de Assis, Florínea, Martinópolis e Paraguaçu Paulista. A pesquisa integrou o “Estudo AGEPEN: Condições de trabalho, saúde mental e sono em policiais penais do Estado de São Paulo” (18,19), que selecionou, por conveniência, quatro penitenciárias com porte semelhante, destinadas exclusivamente a pessoas privadas de liberdade do sexo masculino, para a realização do estudo com esses profissionais.

### Participantes 

A pesquisa foi realizada com todos os policiais penais que preenchiam os critérios de inclusão: ser do sexo masculino e trabalhar há seis meses ou mais como policiais penais nas unidades pesquisadas. Foram considerados como perdas os policiais penais não entrevistados e os que apresentaram registros incompletos sobre as variáveis de estudo. Para o estudo, indivíduos com menos de 12 meses de profissão foram excluídos da análise de dados. A coleta de dados ocorreu ao longo da jornada de trabalho, sendo realizada, no início do expediente, para todas as equipes e turnos, uma explicação dos objetivos da pesquisa, e a seguir, para os que concordaram participar, foi entregue e explicado o Termo de Consentimento Livre e Esclarecido. Os policiais penais que assinaram e devolveram o termo receberam um questionário estruturado e anônimo, juntamente com um envelope pardo, no qual deveriam depositar o material preenchido, que foi recolhido ao final do expediente.

### Variáveis 

A caracterização sociodemográfica foi composta pela idade (contínua), escolaridade (dicotomizada em: ensino médio; e ensino superior: superior, pós-graduação ou mestrado/doutorado) e coabitação (vive com companheira/o: casado ou união estável; vive sem companheira/o: solteiro, viúvo ou divorciado). 

As variáveis independentes foram os aspectos ocupacionais: anos de trabalho como policial penal (contínua e categorizada em ≤15 anos e >15 anos; dicotomizado com base na mediana), o setor de trabalho (dicotomizado em administrativo: sem contato com as pessoas privadas de liberdade [administrativo, informática, recursos humanos, entre outras áreas administrativas]; ou operacional: com contato [portaria, gaiolas, inclusão, disciplina, frota, entre outros setores em contato com as pessoas privadas de liberdade]), turno de trabalho na penitenciária (diarista 8h/dia; plantonista noturno 12h/36h; ou plantonista diarista 12h/36h), exigências mentais e físicas (baixas/moderadas ou altas), presença (sim ou não) de problemas de saúde relacionados ao trabalho como policial penal (alguma doença, acidente, ferimento ou outro problema de saúde) nos 12 meses que antecederam a pesquisa, ter sofrido nos 12 meses que antecederam a pesquisa (sim ou não) violência psicológica (gozação ou situações humilhantes/constrangedoras, assédio moral ou ameaça à integridade física ou familiar) ou física (agressão física, por arma branca ou de fogo), e o grau de satisfação com as condições do trabalho – temperatura, iluminação, ruído, ventilação, higiene e mobiliário (dicotomizadas em ruim; ou regular/boa). 

### Fontes de dados e mensuração

O estresse ocupacional foi mensurado pelo questionário Demand-Control-Support Questionnaire (20). A versão validada e propriedades psicométricas adequadas em português, e utilizada na pesquisa, possui 17 questões, sendo cinco para avaliar a demanda psicológica no trabalho, seis para avaliar o controle sobre o trabalho e seis para avaliar o suporte social (20). No presente estudo, a análise por quadrante foi realizada por meio da soma dos escores de cada dimensão, e posterior divisão em duas categorias a partir da mediana (11,20,21). Desse modo, foram pontos de corte valores maiores que 14 como alta demanda, maiores do que 15 pontos como autocontrole no trabalho, e, maiores que 17 pontos como alto apoio social. Os resultados obtidos foram combinados conforme preconizado, sendo as quatro categorias do modelo demanda-controle: alta exigência (alta demanda e baixo controle), trabalho ativo (alta demanda e alto controle), trabalho passivo (baixa demanda e baixo controle) e baixa exigência (baixa demanda e alto controle). Para a análise, o grupo referência adotado foi o de trabalho de baixa exigência. A dimensão *apoio social* foi dicotomizada em baixo e alto, sendo o alto apoio social considerado como categoria de referência.

### Controle de viés

A formulação do questionário de pesquisa foi precedida por verificação na literatura de instrumentos e questões validadas para a inclusão na pesquisa, tal como a do Demand-Control-Support Questionnaire. As questões que não foram extraídas de instrumentos validados foram apreciadas por um comitê de especialistas em epidemiologia, composto por duas doutorandas em saúde coletiva e cinco docentes, todos doutores em saúde coletiva. A versão final foi aprovada em consenso pelo comitê de especialistas.

Em relação às estratégias da coleta, esta foi precedida por um estudo piloto em uma unidade prisional, no qual foram aprimoradas tais estratégias, para que os policiais penais tivessem segurança em relação ao sigilo e anonimato da pesquisa. A digitação dos instrumentos foi realizada em duplicata no programa Epi Info 3.5.4, com posterior consolidação e correção de eventuais inconsistências. Por fim, as análises estatísticas foram ajustadas por possíveis variáveis de confusão, conforme detalhado nos métodos estatísticos.

### Tamanho do estudo e amostra

O estudo não previu estimativa para tamanho de amostra, visto que buscou entrevistar todos os policiais penais das penitenciárias incluídas. No período de estudo, estavam registrados, nas quatro penitenciárias incluídas, um total de 566 policiais penais. Destes, 213 (37,6%) foram considerados como perdas (82 estavam de licença, 36 policiais penais não foram localizados após três tentativas e 95 recusaram-se a participar do estudo), resultando em uma taxa de resposta de 62,4% (n=353). Ademais, 87 (15,4%) policiais penais realizaram o preenchimento incompleto de uma ou mais variáveis em estudo, sendo, portanto, considerados como perda, e um possuía menos de 12 meses na profissão, sendo excluídos das análises, o que resultou em uma amostra de estudo de 265 (46,8%) policiais penais. 

### Métodos estatísticos

As análises foram realizadas com utilização do programa Statistical Package for the Social Science versão 25.0. Foram apresentadas as frequências absolutas e relativas (variáveis categóricas), bem como média e desvio-padrão (para idade). Objetivando a identificação de possíveis associações que pudessem alterar as análises de fatores ocupacionais e do estresse ocupacional, foi realizada a avaliação da possível associação das variáveis de caracterização em relação às variáveis dependentes. Foi utilizado o teste de chi-quadrado para variáveis categóricas, teste de Kruskal-Wallis para o quadrante demanda-controle e Mann-Whitney para a dicotomização do apoio social (variáveis contínuas com distribuição não paramétrica de acordo com o teste de Shapiro-Wilk). Adicionalmente, foi realizado teste de correlação de Spearman entre as variáveis de caracterização e ocupacionais em relação ao somatório das dimensões demanda, controle e apoio social. Para esta análise, foi considerada a idade em anos (variável contínua), ao passo que as variáveis categóricas foram avaliadas com base na numeração 1 e 2, sendo para a escolaridade ensino médio e ensino superior, e para coabitação, sem companheiro(a) e com companheiro(a), respectivamente.

O efeito das variáveis ocupacionais no estresse ocupacional foi avaliado por meio de modelos de regressão logística multinomial em relação ao modelo demanda-controle e apoio social, com cálculo do valor das razões de chances (*odds ratio*, OR) e intervalos de confiança de 95% (IC95%). Foram realizados modelos brutos (não apresentados) e ajustados por variáveis que apresentam efeito na literatura (idade, escolaridade) (5) ou na presente pesquisa (coabitação) com as variáveis dependentes, bem como a inclusão do apoio social (contínuo) como ajuste para as análises nas quais o modelo demanda-controle foi a variável dependente, e do modelo demanda-controle como ajuste para as análises em que o apoio social foi a variável dependente (16,20). 

## Resultados

O perfil geral dos policiais penais participantes identificou que a maioria possuía 45 anos ou menos (54,7%), um(a) companheiro(a) (86,8%) e nível médio de escolaridade (55,5%). A idade média foi de 44,5 ± 7,9 anos, não havendo diferença na distribuição da idade entre os participantes quando estratificados de acordo com o modelo demanda-controle (p-valor 0,223) ou apoio social (p-valor de 0,487). Em relação à distribuição dos quadrantes, 24,5% dos policiais penais foram classificados como trabalho de alta exigência; 30,9%, trabalho passivo; 20,8%, trabalho ativo; e 23,8%, trabalho de baixa exigência. Em relação ao apoio social, 55,8% dos participantes ficaram com ≤17 e foram classificados como com menor apoio social (Tabela 1). 

**Tabela 1:** Frequências absoluta (n) e relativa (%), e resultado do teste de chi-quadrado, das características sociodemográficas pelas variáveis do estudo. São Paulo, 2019 (n=265)

		Modelo demanda-controle					Apoio social		
Variáveis	Total n	Alta exigência n (%)	Passivo n (%)	Ativo n (%)	Baixa exigência n (%)	p-valor	Baixo n (%)	Alto n (%)	p-valor
Escolaridade						0,106			0,441
Ensino médio	147	33 (22,4)	47 (32,0)	25 (17,0)	42 (28,6)		79 (53,7)	68 (46,3)	
Ensino superior	118	32 (27,1)	35 (29,7)	30 (25,4)	21 (17,8)		69 (58,5)	49 (41,5)	
Coabitação						0,228			0,002
Sem companheiro(a)	35	4 (11,4)	13 (37,2)	7 (20,0)	11 (31,4)		11 (31,4)	24 (68,6)	
Com companheiro(a)	230	61 (26,5)	69 (30,0)	48 (20,9)	52 (22,6)		137 (59,6)	93 (40,4)	

Entre as variáveis sociodemográficas investigadas, ter companheiro apresentou maior frequência de indivíduos classificados com menor apoio social (Tabela 1) e efeito compatível com a diminuição no escore contínuo do apoio social (Tabela 2). O aumento da idade apresentou efeito compatível com a diminuição do escore da demanda de trabalho, enquanto possuir ensino superior apresentou efeito compatível com o aumento do escore da demanda de trabalho e diminuição do escore do apoio social (Tabela 2).

**Tabela 2:** Correlação de Spearman para estimativa do coeficiente de correlação (rô) e p-valor, entre variáveis sociodemográficas e escore contínuo das dimensões demanda, controle e apoio social. São Paulo, 2019 (n=265)

Variáveis	Idade Rô; p-valor	Escolaridade Rô; p-valor	Coabitação Rô; p-valor	Demanda Rô; p-valor	Controle Rô; p-valor
Idade	-				
Escolaridade	-0,112; 0,069	-			
**Coabitação**	0,006; 0,917	0,036; 0,565	-		
Demanda	-0,136; 0,027	0,145; 0,019	0,117; 0,057	-	
Controle	-0,022; 0,719	-0,050; 0,418	-0,063; 0,310	0,115; 0,060	-
Apoio	-0,018; 0,770	-0,126; 0,040	-0,230; <0,001	-0,271; <0,001	0,289; <0,001

Os policiais penais que avaliaram como ruim o nível de satisfação com a temperatura e ventilação do ambiente de trabalho apresentaram maior frequência de trabalho de alta exigência (Tabela 3), com correlação entre o nível de satisfação ruim e a menor pontuação no escore de controle sobre o trabalho (Tabela 2). O menor apoio social foi observado principalmente nos indivíduos com mais de 15 anos na profissão, que relataram ter sofrido violência física e psicológica e que avaliaram como ruim o nível de satisfação sobre condições do ambiente de trabalho.

**Tabela 3:** Frequências absolutas (n) e relativas (%) das características ocupacionais pelas variáveis do estudo. São Paulo, 2019 (n=265)

	Modelo demanda-controle					Apoio social	
Variáveis	Alta exigência n (%)	Passivo n (%)	Ativo n (%)	Baixa exigência n (%)		Baixo n (%)	Alto n (%)
**Tempo de trabalho como policial penal**							
≤15 anos	29 (25,4)	35 (30,7)	24 (21,1)	26 (22,8)		54 (47,4)	60 (52,6)
>15 anos	36 (23,9)	47 (31,1)	31 (20,5)	37 (24,5)		94 (62,3)	57 (37,7)
**Setor de trabalho**							
Operacional	51 (26,2)	65 (33,3)	36 (18,4)	43 (22,1)		113 (57,9)	82 (42,1)
Administrativo	14 (20,0)	17 (24,3)	19 (27,1)	20 (28,6)		35 (50,0)	35 (50,0)
**Turno de trabalho**							
Plantonista noturno	23 (26,7)	33 (38,4)	4 (4,7)	26 (30,2)		49 (57,0)	37 (43,0)
Plantonista diurno	28 (23,5)	34 (28,6)	26 (21,8)	31 (26,1)		66 (55,5)	53 (44,5)
Diarista	14 (23,3)	15 (25,0)	25 (41,7)	6 (10,0)		33 (55,0)	27 (45,0)
**Exigências físicas**							
Altas	57 (28,7)	49 (24,6)	52 (26,1)	41 (20,6)		119 (59,8)	80 (40,2)
Baixas/moderadas	8 (12,1)	33 (50,0)	3 (4,5)	22 (33,4)		29 (43,9)	37 (56,1)
**Exigências mentais**							
Altas	13 (36,1)	2 (5,5)	11 (30,6)	10 (27,8)		22 (61,1)	14 (38,9)
Baixas/moderadas	52 (22,8)	80 (34,9)	44 (19,2)	53 (23,1)		126 (55,0)	103 (45,0)
**Problema de saúde relacionada ao trabalho**							
Sim	17 (34,7)	13 (26,5)	9 (20,4)	10 (20,4)		31 (63,3)	18 (36,7)
Não	48 (22,2)	69 (31,9)	46 (21,3)	53 (24,6)		117 (54,2)	99 (45,8)
**Violência psicológica**							
Sim	26 (32,5)	17 (21,3)	23 (28,7)	14 (17,5)		60 (75,0)	20 (25,0)
Não	39 (21,1)	65 (35,1)	32 (17,3)	49 (26,5)		88 (47,6)	97 (52,4)
**Violência física**							
Sim	9 (36,0)	5 (20,0)	4 (16,0)	7 (28,0)		19 (76,0)	6 (24,0)
Não	56 (23,3)	77 (32,1)	51 (21,3)	56 (23,3)		129 (53,8)	111 (46,2)
**Satisfação com a temperatura do ambiente de trabalho**							
Ruim	35 (35,4)	37 (37,4)	16 (16,2)	11 (11,0)		67 (67,7)	32 (32,3)
Boa/regular	30 (18,1)	45 (27,1)	39 (23,5)	52 (31,3)		81 (48,8)	85 (51,2)
**Satisfação com a iluminação do ambiente de trabalho**							
Ruim	15 (41,7)	11 (30,6)	7 (19,4)	3 (8,3)		29 (80,6)	7 (18,4)
Boa/regular	50 (21,8)	71 (31,0)	48 (21,0)	60 (26,2)		119 (52,0)	110 (48,0)
**Satisfação com o ruído do ambiente de trabalho**							
Ruim	32 (32,7)	35 (35,7)	15 (15,3)	16 (16,3)		76 (77,6)	22 (22,4)
Boa/regular	33 (19,8)	47 (28,1)	40 (24,0)	47 (28,1)		72 (43,1)	95 (56,9)
**Satisfação com a ventilação do ambiente de trabalho**							
Ruim	32 (32,3)	36 (36,4)	20 (20,2)	11 (11,1)		71 (71,7)	28 (28,3)
Boa/regular	33 (19,9)	46 (27,7)	35 (21,1)	52 (31,3)		77 (46,4)	89 (53,6)
**Satisfação com a higiene do ambiente de trabalho**							
Ruim	17 (34,7)	14 (28,6)	10 (20,4)	8 (16,3)		36 (73,5)	13 (26,5)
Boa/regular	48 (22,2)	68 (31,5)	45 (20,8)	55 (25,5)		112 (51,9)	104 (48,1)
**Satisfação com a mobília do ambiente de trabalho**							
Ruim	27 (34,6)	26 (33,3)	16 (20,6)	9 (11,5)		61 (78,2)	17 (21,8)
Boa/regular	38 (20,3)	56 (29,9)	39 (20,9)	54 (28,9)		87 (46,5)	100 (53,5)

Os modelos ajustados identificaram que apenas avaliação ruim para a satisfação com a temperatura e ventilação apresentaram efeito compatível com o aumento do trabalho de alta exigência (Tabela 4). O menor apoio social se associou com maior tempo como policial penal (mais de 15 anos), relato de violência psicológica e física, e com nível de satisfação ruim com relação a iluminação, ruído, ventilação e mobília do ambiente de trabalho (Tabela 5).

**Tabela 4:** Razões de chances ajustada (OR) e intervalos de confiança de 95% (IC95%) do modelo demanda–controle pelas variáveis do estudo. São Paulo, 2019 (n=265)

Variáveis	Alta exigência OR (IC95%)	Passivo OR (IC95%)	Ativo OR (IC95%)
**Tempo de trabalho como policial penal**			
≤15 anos	1,00	1,00	1,00
>15 anos	0,57 (0,22; 1,49)	0,62 (0,26; 1,49)	1,06 (0,40; 2,81)
**Setor de trabalho**			
Operacional	1,54 (0,66; 3,59)	1,69 (0,77; 3,68)	0,89 (0,40; 1,98)
Administrativo	1,00	1,00	1,00
**Turno de trabalho**			
Plantonista noturno	0,36 (0,11; 1,20)	0,48 (0,16; 1,47)	0,04 (0,01; 0,15)
Plantonista diurno	0,31 (0,09; 1,00)	0,39 (0,13; 1,21)	0,18 (0,06; 0,53)
Diarista	1,00	1,00	1,00
**Exigências físicas**			
Altas	2,41 (0,91; 6,38)	0,56 (0,26; 1,18)	7,41 (2,01; 27,38)
Baixas/moderadas	1,00	1,00	1,00
**Exigências mentais**			
Altas	1,27 (0,46; 3,46)	0,12 (0,02; 0,59)	1,40 (0,53; 3,74)
Baixas/moderadas	1,00	1,00	1,00
**Problema de saúde relacionado ao trabalho**			
Sim	1,37 (0,53; 3,52)	0,80 (0,31; 2,05)	0,84 (0,30; 2,33)
Não	1,00	1,00	1,00
**Violência psicológica**			
Sim	1,03 (0,43; 2,47)	0,52 (0,22; 1,25)	1,78 (0,76; 4,21)
Não	1,00	1,00	1,00
**Violência física**			
Sim	0,99 (0,31; 3,14)	0,45 (0,13; 1,56)	0,48 (0,13; 1,83)
Não	1,00	1,00	1,00
**Satisfação com a temperatura do ambiente de trabalho**			
Ruim	4,88 (2,02; 11,81)	3,80 (1,67; 8,66)	1,86 (0,74; 4,63)
Boa/regular	1,00	1,00	1,00
**Satisfação com a iluminação do ambiente de trabalho**			
Ruim	3,83 (0,97; 15,11)	2,53 (0,65; 9,89)	2,21 (0,52; 9,34)
Boa/regular	1,00	1,00	1,00
**Satisfação com o ruído do ambiente de trabalho**			
Ruim	1,55 (0,67; 3,53)	1,55 (0,72; 3,33)	0,83 (0,34; 2,01)
Boa/regular	1,00	1,00	1,00
**Satisfação com a ventilação do ambiente de trabalho**			
Ruim	3,12 (1,31; 7,41)	3,07 (1,36; 6,89)	2,23 (0,92; 5,39)
Boa/regular	1,00	1,00	1,00
**Satisfação com a higiene do ambiente de trabalho**			
Ruim	1,63 (0,59; 4,45)	1,13 (0,42; 3,00)	1,40 (0,49; 4,04)
Boa/regular	1,00	1,00	1,00
**Satisfação com a mobília do ambiente de trabalho**			
Ruim	2,30 (0,91; 5,86)	2,03 (0,84; 4,91)	1,88 (0,72; 4,92)
Boa/regular	1,00	1,00	1,00

**Tabela 5:** Razões de chances ajustada (OR) e intervalos de confiança de 95% (IC95%) da dimensão *apoio social* pelas variáveis do estudo. São Paulo, 2019 (n=265)

Variáveis	Baixo apoio social OR (IC95%)
**Tempo de trabalho como policial penal**	
≤15	1,00
>15	2,59 (1,33; 5,08)
**Setor de trabalho**	
Operacional	1,18 (0,65; 2,17)
Administrativo	1,00
**Turno de trabalho**	
Plantonista noturno	1,42 (0,66; 3,08)
Plantonista diurno	1,21 (0,61; 2,43)
Diarista	1,00
**Exigências físicas**	
Altas	1,64 (0,85; 3,15)
Baixas/moderadas	1,00
**Exigências mentais**	
Altas	1,11 (0,50; 2,47)
Baixas/moderadas	1,00
**Problema de saúde relacionado ao trabalho**	
Sim	1,09 (0,54; 2,20)
Não	1,00
**Violência psicológica**	
Sim	2,93 (1,55; 5,53)
Não	1,00
**Violência física**	
Sim	2,84 (1,02; 7,93)
Não	1,00
**Satisfação com a temperatura do ambiente de trabalho**	
Ruim	1,69 (0,94; 3,03)
Boa/regular	1,00
**Satisfação com a iluminação do ambiente de trabalho**	
Ruim	2,80 (1,11; 7,08)
Boa/regular	1,00
**Satisfação com o ruído do ambiente de trabalho**	
Ruim	3,94 (2,15; 7,21)
Boa/regular	1,00
**Satisfação com a ventilação do ambiente de trabalho**	
Ruim	2,15 (1,20; 3,85)
Boa/regular	1,00
**Satisfação com a higiene do ambiente de trabalho**	
Ruim	1,89 (0,90; 3,94)
Boa/regular	1,00
**Satisfação com a mobília do ambiente de trabalho**	
Ruim	2,92 (1,52; 5,60)
Boa/regular	1,00

## Discussão

Entre as variáveis ocupacionais investigadas, avaliar como ruim o nível de satisfação com a temperatura e ventilação do ambiente de trabalho apresentou efeito compatível com o aumento de chance para trabalho de alta exigência e passivo. Ter maiores exigências mentais apresentou efeito compatível com a menor chance para trabalho passivo. Em comparação com policiais penais que trabalhavam em regime diarista (8 horas por dia), aqueles em plantão (12 horas) noturno e diurno apresentaram efeito compatível com menor chance de trabalho ativo. Relatar ter maiores exigências físicas no trabalho apresentou efeito compatível com maior chance para trabalho ativo. Por fim, os policiais penais com mais de 15 anos de profissão, relato de violência física e psicológica, e aqueles com nível de satisfação ruim em relação a algumas condições do ambiente de trabalho apresentaram efeito compatível com maior chance para a presença de menor apoio social.

Em relação ao trabalho, o ponto de corte para alta demanda (˃14) ficou próximo ao encontrado em policiais (˃14) (22), técnicos administrativos (˃14) (23) e professores (˃15) (16, 23). O ponto de corte para baixo controle do trabalho (≤15) foi um pouco mais elevado do que o observado em outros profissionais de segurança (≤11 policiais) (22) e inferior ao relatado em profissionais da área educacional (≤17) (16). Dessa forma, pode-se deduzir que, embora a organização do trabalho seja distinta entre essas profissões, os policiais penais avaliam a carga de trabalho de maneira semelhante à percebida por outros profissionais. No entanto, o controle sobre o próprio processo de trabalho parece ser mais limitado entre os policiais penais, possivelmente devido à natureza organizada em níveis ascendentes de comando e aos riscos inerentes ao exercício profissional (1). 

O trabalho em regime de plantão, independentemente do turno, apresentou efeito compatível com a menor chance para trabalho ativo. Esses achados podem ser explicados, mesmo que em parte, pela teoria do efeito de “prisionização” (5), segundo a qual a exposição contínua, sem a possibilidade de descanso no dia seguinte ao expediente, como ocorre com os que trabalham em regime de plantão, pode intensificar os efeitos da carga de trabalho sobre a saúde mental. De acordo com essa teoria, o trabalho em regime de plantão pode permitir, com folga no dia seguinte ao dia trabalhado, um maior desligamento das atividades laborais e recuperação dos efeitos gerados pelas demandas de trabalho.

A associação encontrada entre o trabalho de alta exigência e a pior grau de satisfação profissional pode ser decorrente do fato de que essa condição de trabalho, na qual há exacerbação dos efeitos negativos das demandas, gera uma avaliação negativa por parte do profissional a respeito do seu processo de trabalho e das perspectivas futuras em relação à vida laboral, o que pode levar à sensação de incapacidade para o desempenho de suas funções (24). Nesse contexto, em diversas instituições prisionais, identifica-se a influência do estresse ocupacional na redução da satisfação no trabalho (25). Embora estes efeitos estejam bem descritos na literatura (26), no presente estudo apenas o nível de satisfação ruim com a temperatura e iluminação apresentaram efeito compatível com maior estresse ocupacional. Uma possível explicação para esse achado é a abordagem adotada no estudo, que analisou individualmente cada aspecto do ambiente de trabalho, o que possibilitou a identificação dos fatores mais impactantes na percepção dos policiais penais investigados.

A organização do processo de trabalho nas penitenciárias pode, pelo menos em parte, justificar a associação entre altas exigências físicas e o trabalho ativo, uma vez que os trabalhos físicos são exercidos, em sua maioria, por policiais penais em setores operacionais. Essa característica faz com que estes policiais penais, ao mesmo tempo que realizam mais atividades físicas, também estejam mais expostos a pessoas privadas de liberdade, o que pode acentuar a percepção das demandas do trabalho (5,24). Essa exposição também pode proporcionar maior controle sobre a forma como desempenham suas funções, em contraste com os policiais penais em funções administrativas. Embora o setor de trabalho tenha forte influência na jornada laboral, outro aspecto importante também atrelado a essa variável é o nível de exposição a situações de conflito, a necessidade de se realizar dupla função (educação e manutenção da ordem) e maior contato com as pessoas privadas de liberdade, o que pode aumentar o estresse ocupacional (27,28).

A percepção de maiores exigências mentais apresentou efeito compatível com a menor chance para a classificação como trabalho passivo. Tal associação parece se justificar, novamente, pelo efeito de “prisionização” (5), agravado pela característica do processo de trabalho, no qual os policiais penais em funções de coordenação se inserem em uma rotina bem estabelecida, que não fornece possibilidade de adaptação do modo de trabalho (29). No entanto, não foram encontrados estudos que tenham se debruçado sobre a organização de trabalho e funções que policiais penais desempenham e os efeitos destas variáveis com a demanda e controle do processo de trabalho.

Em relação ao apoio social, os efeitos identificados parecem ir na direção de que os policiais penais com menor apoio social sentem mais as demandas físicas, as violências e as condições do ambiente de trabalho. Confirma-se, assim, o papel atenuador que o apoio social possui, tanto frente ao estresse laboral (elevadas demandas e baixo controle) como a outras variáveis ocupacionais mais gerais (16,20,30). Esses achados justificam a aparente maior exaustão identificada em policiais penais, em comparação a outras profissões, sendo necessário, além da análise do estresse ocupacional, considerar as interferências de outras variáveis individuais, como sexo e idade, além de variáveis relacionadas à organização do trabalho, como carga horária, traumas e realização de funções com maior exposição a conflitos (28,31).

Diversas são as teorias que se propõem a explicar os mecanismos envolvidos neste processo, sendo que, em linhas gerais, o maior apoio social pode diminuir diretamente o efeito de demandas instrumentais de trabalho, conflitos afetivos e relativos ao equilíbrio do trabalho com a vida pessoal (30). Além disso, o apoio social apresenta efeitos indiretos, aumentando a resiliência e outros mecanismos de enfrentamento de situações estressantes (32). Estes efeitos acabam se estendendo em rede, gerando diminuição do estresse ocupacional e do burnout, além de auxiliar em recursos individuais, como autonomia e competências laborais, o que em conjunto pode melhorar a autonomia e engajamento no trabalho, promovendo melhores resultados, que retroalimentam a cadeia por meio de melhoria do apoio social (30). 

Não obstante, a redução do estresse ocupacional transcende as dimensões da saúde mental, influenciando outros comportamentos importantes para um estilo de vida saudável, como o sono (18) e a alimentação (33), o que reforça a importância de ações que visem ao seu controle ou atenuação. Assim, o aumento do apoio social se mostra como eficiente medida para a redução do estresse ocupacional e melhoria da qualidade de vida (34). Em razão da complexidade do problema, faz-se necessária a adoção de mudanças sistemáticas, haja vista que mudanças pontuais de melhoria de relacionamento e da hierarquia apresentam pouco impacto sobre o estresse ocupacional, devendo tais mudanças serem acompanhadas da diminuição da carga de trabalho (35).

Este estudo possui como principais limitações o recorte transversal, que não permite a avaliação de causalidade ou direcionalidade entre as variáveis investigadas, bem como o fato de os dados serem relativos apenas a policiais penais de um sexo (masculino) e de apenas uma Unidade da Federação, o que pode limitar a generalização dos achados para outras populações. Além disso, considera-se a extrapolação dos resultados limitada, mesmo para as penitenciárias incluídas na pesquisa, devido ao elevado percentual de perdas. Contudo, ressaltam-se, como pontos fortes do estudo, o uso de escalas validadas internacionalmente e, apesar das perdas, o elevado número de policiais penais que responderam a todas as questões da pesquisa, que propiciaram um estudo abrangente e com análises estatísticas ajustadas por fatores de confusão. 

Com base nos achados, reforça-se a importância de estratégias que possam promover maior controle sobre o trabalho, diminuição das demandas e fortalecimento das redes de apoio social entre os profissionais, como forma de atenuar os impactos adversos à saúde provocados pelo estresse ocupacional.

## Data Availability

O banco de dados utilizado na pesquisa pode ser obtido mediante envio de solicitação e justificativa ao autor correspondente.
